# Haematopoietic stem cell-derived immune cells have reduced X chromosome inactivation skewing in systemic lupus erythematosus

**DOI:** 10.1136/ard-2024-225585

**Published:** 2024-06-27

**Authors:** Amy L Roberts, Alessandro Morea, Ariella Amar, Magdalena West, Sarah Karrar, Rhiannon Lehane, Philip Tombleson, Deborah Cunningham Grahman, John A Reynolds, Chloe C Y Wong, David L Morris, Kerrin S Small, Timothy J Vyse

**Affiliations:** 1 Twin Research and Genetic Epidemiology, King's College London, London, UK; 2 Foundation Institute of Molecular Oncology, IFOM, Milano, Italy; 3 Department of Medical and Molecular Genetics, King's College London, London, UK; 4 Institute of Inflammation and Ageing, University of Birmingham, Birmingham, UK; 5 Institute of Psychiatry, Psychology & Neuroscience, King's College London, London, UK; 6 Medical and Molecular Genetics, King's College London, London, UK; 7 Genetics, King's College London, London, UK

**Keywords:** Lupus Erythematosus, Systemic, Autoimmune Diseases, Polymorphism, Genetic

## Abstract

**Objectives:**

Systemic lupus erythematosus (SLE) shows a marked female bias in prevalence. X chromosome inactivation (XCI) is the mechanism which randomly silences one X chromosome to equalise gene expression between 46, XX females and 46, XY males. Though XCI is expected to result in a random pattern of mosaicism across tissues, some females display a significantly skewed ratio in immune cells, termed XCI-skew. We tested whether XCI was abnormal in females with SLE and hence contributes to sexual dimorphism.

**Methods:**

We assayed XCI in whole blood DNA in 181 female SLE cases, 796 female healthy controls and 10 twin pairs discordant for SLE. Using regression modelling and intra-twin comparisons, we assessed the effect of SLE on XCI and combined clinical, cellular and genetic data via a polygenic score to explore underlying mechanisms.

**Results:**

Accommodating the powerful confounder of age, XCI-skew was reduced in females with SLE compared with controls (p=1.3×10^−5^), with the greatest effect seen in those with more severe disease. Applying an XCI threshold of >80%, we observed XCI-skew in 6.6% of SLE cases compared with 22% of controls. This difference was not explained by differential white cell counts, medication or genetic susceptibility to SLE. Instead, XCI-skew correlated with a biomarker for type I interferon-regulated gene expression.

**Conclusions:**

These results refute current views on XCI-skew in autoimmunity and suggest, in lupus, XCI patterns of immune cells reflect the impact of disease state, specifically interferon signalling, on the haematopoietic stem cells from which they derive.

**WHAT IS ALREADY KNOWN ON THIS TOPIC**

- Increased prevalence of skewed X chromosome inactivation (XCI-skew) in females has been reported for numerous autoimmune diseases and it is hypothesised to contribute to disease development and sex biases in the prevalence. This hypothesis has not been robustly tested in systemic lupus erythematosus (SLE).

**WHAT THIS STUDY ADDS**

- XCI-skew is reduced in SLE cases compared with healthy controls. This effect is driven by disease progression and likely reflects the impact of disease state, driven by chronic IFN-signalling, on haematopoietic stem and progenitor cells.

**HOW THIS STUDY MIGHT AFFECT RESEARCH, PRACTICE OR POLICY**

- Our work has implications for our understanding of immune system ageing in individuals with autoimmune diseases.

## Introduction

 Systemic lupus erythematosus (SLE) is a multisystem autoimmune disease, which presents with an incompletely understood sexual dimorphism. Females represent ~90% of cases and SLE is a leading cause of death in females aged under 34 years of age.^1^ Whereas hormonal factors were initially thought to explain the sex bias, attention has recently focused on the sex chromosomes as contributory factors.^2^ Strong epidemiological evidence supports X chromosome dosage as a substantial risk factor: males with Klinefelter’s syndrome (47, XXY) have a 14-fold increased prevalence of SLE compared with 46, XY males^3^; females with Turner’s syndrome (45, XO) have a lower risk, and 47, XXX females have a higher risk, compared with 46, XX females.^4 5^

There is complexity when interpreting the role of the X chromosome in disease. In mammals, X chromosome inactivation (XCI) ensures that only one X chromosome is active within each cell—any additional Xs are transcriptionally shut down, resulting in a functionally inactive chromosome (Xi). This process evolved to equalise the gene expression between 46, XX females and 46, XY males.^6^ In humans, during development the choice of which X is silenced in each cell is random and the Xi status is then clonally inherited by any daughter cells. Therefore, despite the karyotypic differences in sex chromosomes, every mammalian cell has only one active X chromosome.

Though XCI is expected to result in a random pattern of mosaicism across tissues, a great deal of variation has been observed across humans, with some females displaying a significantly unbalanced ratio, termed XCI-skew.^7^,^9^ The prevalence of XCI-skew in blood increases with age and represents a common age-acquired phenotype in females: one-third of females over 60 years have an XCI ratio in blood of 80:20 or greater.^10^ Age-acquired XCI-skew in blood is hypothesised to arise from long-term changes to the underlying haematopoietic stem and progenitor cells (HSPCs), such as stem cell exhaustion or clonal expansion, and is not thought to reflect short-term fluctuations in HSPC activity.^11 12^ Further, it has been hypothesised that XCI-skew of immune cells could play a causal role in the development of autoimmune disease.^13^ There is some evidence that the prevalence of XCI-skew in blood cells is increased in autoimmunity, including autoimmune thyroid disease,^14^,^16^ rheumatoid arthritis^16 17^ and systemic sclerosis.^18 19^ However, this is not consistent across autoimmune conditions.^13 20 21^

Conversely, autoimmune disease progression could also influence XCI-skew in blood cells. Given the selection of the Xi is stable across cell divisions, the XCI ratio of the peripheral immune cells must reflect that of the HSPCs from which they derive. HSPCs can be directly influenced by cytokine signalling,^22 23^ including interferon (IFN)-α which is a key cytokine in the pathogenesis of SLE.^24^ In a mouse model of SLE, inflammation resulted in significantly expanded HSPCs with increased self-renewal capacity.^25^ In humans, the dysfunction of immune cells in SLE can be traced back to HSPCs, where CD34+ HSPCs from SLE patients with severe disease showed enhanced proliferation and cell differentiation, together with a distinct gene expression signature.^26^ The impact of the cytokine environment of SLE on HSPCs could, therefore, be reflected in the XCI measures of the HSPC-derived immune cells.

Despite SLE having one of the most marked female sex predilections across all autoimmune conditions, and disease pathogenesis being driven by HSPC-derived immune cells, the role of XCI-skew of blood cells in SLE has yet to be established. We assayed XCI from whole blood in 181 female patients with SLE and 796 female controls from the TwinsUK population cohort, and combined clinical, cellular and genetic data to robustly investigate XCI in SLE.

## Methods

### SLE cohort

Archival DNA samples derived from whole blood (collected 2013–2019) were collected from 260 female patients with SLE at Guy’s and St Thomas’ Hospital, Birmingham Hospital, and Maidstone Hospital, and assayed for XCI. This resulted in 181 informative samples from unrelated individuals, with a median age of 50 years (table 1). All volunteers met the 1997 American College of Rheumatology criteria for SLE.^27^

**Table 1:** Descriptive of SLE cases and control cohorts

	Controls	SLE cases
Sample size	796	181
Age (median (range))	59.5 (20–74)	50 (18–79)
Years of disease (median (range))	–	12 (1–47)
Age at diagnosis (median (range))	–	32 (5–75)
Hydroxychloroquine (n (%))	–	112 (61.9)
Methotrexate (n (%))	–	25 (13.8)
Biologics (n (%))	–	11 (9.1)
Azathioprine or mycophenolate (n (%))	–	42 (23.2)
With renal disease (n (%))	–	36 (20.4)
With genotype data (n (%))	750 (94.2)	149 (82.3)
With full blood count data (n (%))	436 (54.8)	42 (23.2)
XCI-skewing (mean)	0.20	0.14
XCI-skewing>80 (n (%))	175 (22.0)	12 (6.6)

SLEsystemic lupus erythematosusXCIX chromosome inactivation

### Twins UK cohort

Archival DNA samples derived from whole blood (collected 1997–2017) were selected from individuals of the TwinsUK population cohort.^28^ 2382 samples were assayed for XCI, which resulted in 1575 informative samples, as described previously.^8^ Individuals with self-reported SLE, as well as their co-twins or self-reported prior treatment with immunosuppressive medication, were excluded. Next, one individual from each twin pair was selected at random resulting in a cohort of 796 unrelated individuals, with a median age of 59.5 (table 1). During this sample selection process, we identified 10 pairs of SLE-discordant twins which were used in a follow-up twin analysis (see below).

### The human androgen receptor assay

The human androgen receptor assay (HUMARA) method is a robust assay used extensively to measure XCI, which combines methylation-sensitive restriction enzyme digest and amplification of a highly polymorphic (CAG)n repeat in the first exon of the X-linked *AR* gene.^29^ The method used was exactly as described previously,^8^ using 625 ng of genomic DNA and processed on an ABI 3730xl. using the GeneScan 500 LIZ size standard.

### Calculation of XCI

Data from the fragment analysis were analysed using the Microsatellite Analysis Software available on the ThermoFisher Cloud. The XCI status was calculated in each of the triplicates as follows:

- Allele Ratio Mock Digestion (Rm)=allele 1 peak height/allele 2 peak height.
- Allele Ratio *HpaII* Digestion (Rh)=allele 1 peak height/allele 2 peak height.
- Normalised ratio (Rn)=Rh/Rm.
- XCI percentage=[Rn/(Rn+1)]×100.

The SD and mean across the triplicates were used to calculate a coefficient of variation (CV) and samples with CV>0.15 were excluded from downstream analysis. The mean XCI percentage (0%–100%) was calculated for each sample, where 50% is a perfectly balanced XCI. The directionality of XCI away from 50% is uninformative (eg, both 0% and 100% are considered equal). Therefore, the XCI values are collapsed to a range of 50%–100% to create a continuous variable termed XCI-skew.

### Whole blood count data

Whole blood count data obtained from standard Coulter-based clinical testing were date-matched to the XCI DNA sample, consisting of counts for white cell count (WCC), monocytes, lymphocytes and neutrophils. The proportion of lymphocytes was calculated by dividing the lymphocyte count by WCC, and monocyte-to-lymphocyte ratio and neutrophil-to-lymphocyte ratio were calculated by dividing the monocyte or neutrophil count, respectively, by lymphocyte count.

### Medication use

Questionnaire data were used to match current medication use to the date of the blood sample for SLE patients. For each of hydroxychloroquine, methotrexate, biologics, azathioprine/mycophenolate, a categorical variable was created where healthy controls were coded as 0, SLE cases without medication use as 1, and SLE cases being treated with the medication as 2.

### Renal disease

Questionnaire data were used to assess the history of renal disease (by ACR criteria) in SLE patients. A categorical variable was created where healthy controls were coded as 0, SLE cases without renal disease as 1 and SLE cases with renal disease as 2.

### SLE polygenic score

A polygenic score (PGS) which captures SLE genetic susceptibility comprising 133 autosomal SNPs (MAF>1%) was used. The PGS model assumes an additive contribution of all SNPs, weighted by their effect sizes. However, skewed XCI will affect this additive assumption for X-linked SNPs, therefore, X-linked SNPs were excluded. Plink2 was used to calculate the SLE-PGS using genome-wide genotype data using the King’s College London CREATE system.^30^ 94.2% and 82.3% of the TwinsUK controls and SLE samples had available genotype data, respectively (table 1). Samples were excluded if they were >3 s.d. away from the mean of heterozygosity across all SNPs.

### Soluble SIGLEC-1 data

Soluble SIGLEC-1 (sSIGLEC-1) concentrations were measured using a non-isotopic time-resolved fluorescence assay based on the dissociation-enhanced lanthanide fluorescent immunoassay technology (PerkinElmer) in plasma samples from 304 SLE cases, as previously described.^31^ sSIGLEC-1 was measured in duplicate and individuals with a CV>0.3 were removed, together with individuals of non-European ancestry, resulting in a dataset of n=299. Patients were divided into groups based on sSIGLEC-1 serum level centiles (<50 th centile, 51st–74th centile, 75th–95th centile and >95 th centile). Of these, 41 individuals had matched XCI data and were used in the analyses.

### Discordant twins

Questionnaire data were used to identify female twin pairs discordant for SLE (n=10 pairs; DZ=6; MZ=4) based on self-reported doctor’s diagnoses. DNA samples from twin pairs were date matched, and therefore, the XCI measures were perfectly matched for age.

### Statistical analysis

For all linear and logistic regression models, XCI-skew was used as the dependent variable and age was included as a covariate. Results were quantified with effect sizes or ORs and 95% CIs. To assess the effects of each of the blood count variables on the disease associations in turn, linear regression models were constructed with XCI-skew as the dependent variable and the cell count as an independent variable (model 1). Residuals from model 1 were used as the dependent variable in a second model with SLE status as an independent variable and age as a covariate. For the sSIGLEC-1 analysis, an additional linear regression model was used with age as an interaction term. For discordant twin analyses, XCI-skew was compared using a one-sided paired sample Wilcoxon test. A p<0.05 was considered significant unless otherwise stated due to multiple testing correction using Bonferroni correction. All analyses were carried out using R V.4.1.1.

## Results

### XCI-skew is reduced in female SLE cases compared with female healthy controls

**Figure 1:**
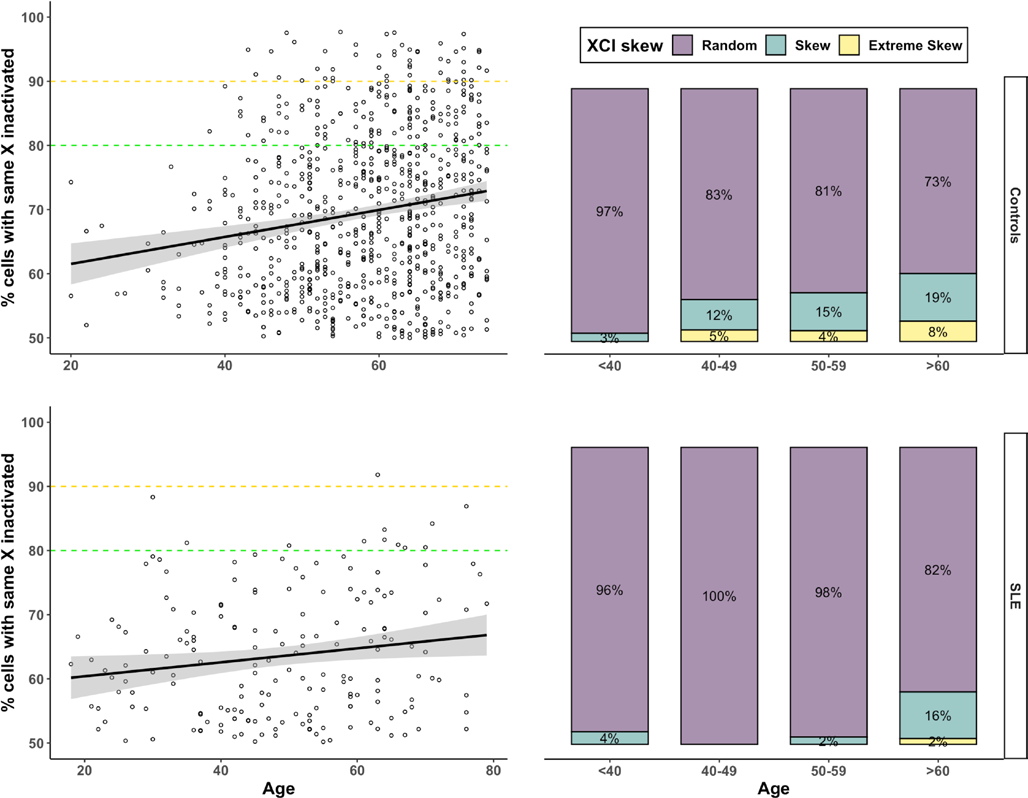
XCI-skewing in SLE and controls The correlation between XCI (y-axis) and age (x-axis) is shown in panels on the left and the proportions of individuals (y-axis) with random (50%–79%), skewed (80%–89%) and extremely skewed (>90%) XCI across increasing age groups (x-axis) are shown in panels on the right. Controls (n=796) are in the upper panels and SLE cases (n=181) are in the lower panels. SLE, systemic lupus erythematosus; XCI, X chromosome inactivation.

We quantified the degree of XCI-skew from 0 (representing 50:50 ratio) to 0.5 (representing a 100:0 ratio) across 181 SLE cases and 796 healthy controls from the TwinsUK population cohort (table 1). We found XCI-skew was positively correlated with age in the SLE cohort (p=0.027, r=0.14; figure 1), as previously described in healthy cohorts.^8 10 32^

However, using a linear regression model with the degree of XCI-skew as the dependent variable, and controlling for age as a covariate, we observed SLE status to be significantly and inversely correlated with XCI-skew (beta=−0.044; p=1.33×10^−5^). To ensure the case–control differences in XCI were not driven by the differences in the age distribution between the cohorts (table 1), we stratified the samples into four age groups, defined as under 40 years of age (yrs), 40–49 years, 50–59 years and over 60 years and applied the same linear regression model within each group (table 2). SLE status was significantly associated with reduced XCI-skew in 40–49 years (p=9.4×10^−4^) and 50–59 years (p=9.8×10^−4^), after Bonferroni correction (alpha=0.0125), and nominally significant in the over 60 years group (p=0.034; online supplemental figure S1). We saw no association in the under 40s group (p=0.61), which may reflect the low frequency of age-associated XCI-skew within this age group in both cases and controls.

**Table 2:** Case–control analysis within age groups

	Controls (n (%))	SLE cases (n (%))	P value	Beta
All samples	796 (100)	181 (100)	1.33×10^–5^	−0.044
40 and under	31 (3.9)	47 (26.0)	0.61	0.010
40s	133 (16.7)	43 (23.8)	0.00094*	−0.065
50s	234 (29.4)	40 (22.1)	0.00098*	−0.064
60 and over	398 (50.0)	51 (28.2)	0.034†	−0.040

* nominally significantsignificant after Bonferroni correction.

† significant after Bonferroni correction nominally significant.

SLEsystemic lupus erythematosus

We also defined XCI-skew (XCI≥80) and extreme XCI-skew (XCI≥90) as binary variables and used logistic regression models to assess their relationship with SLE. We confirmed that SLE status was associated with reduced odds of both XCI-skew, p=0.001; OR=0.90 (0.84–0.96) and extreme XCI-skew, p=0.024; OR=0.96 (0.92–0.99). In the SLE cohort, 6.6% have XCI-skew, and just one individual, equivalent to 0.55%, had extreme skew. A marked contrast with 22% and 6% of control samples, respectively, in these groups (figure 1; table 1).

### Replication using an intra-twin model of SLE discordant twins

**Figure 2:**
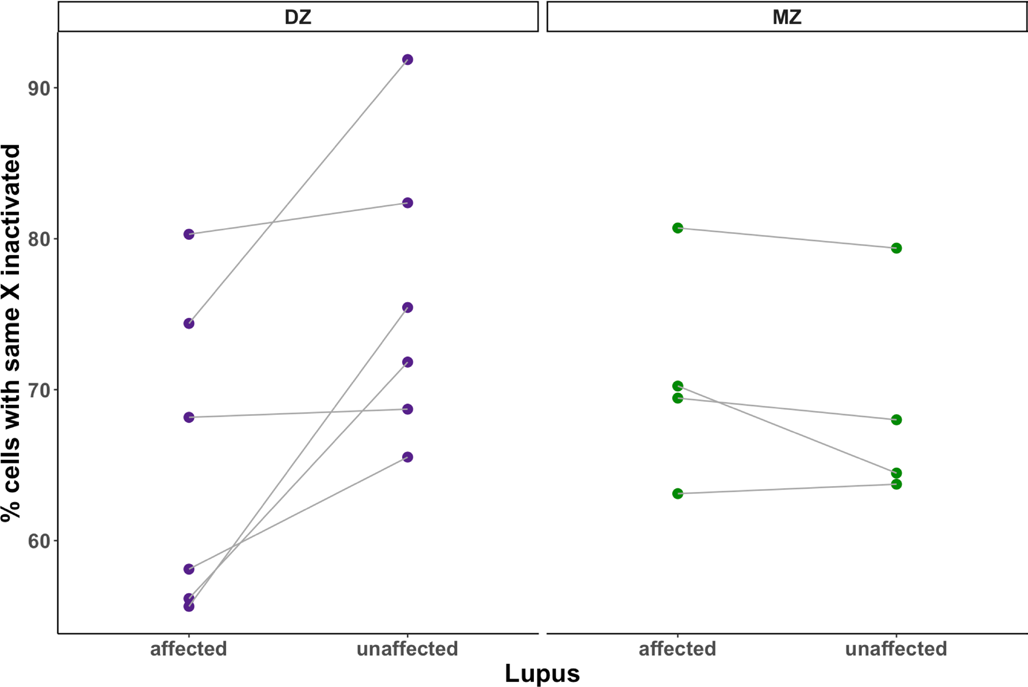
XCI-skewing in a discordant twin study using age-matched twin pairs discordant for SLE (N_pairs_ =10), disease status is associated with decreased XCI skewing in the intratwin analysis of DZ twins (one-sided paired samples Wilcoxon test; p=0.016) but not MZ twins (one-sided paired samples Wilcoxon test; p=0.94). DZ, dizygotic; MZ, monozygotic; SLE, systemic lupus erythematosus; XCI, X chromosome inactivation.

Using twin pairs discordant for SLE, we next assessed whether XCI-skew was reduced in the affected twin compared with their unaffected cotwin and found a nominally significant association (p=0.080). Analysing the dizygotic (DZ) and monozygotic (MZ) twins separately, we observed the association was driven by differences between the discordant DZ twins (p=0.016, n=6; figure 2), whereas no effect was seen between the discordant MZ twins (p=0.94, n=4, figure 2), suggesting potential confounding genetic factors.

### SLE severity further reduces XCI-skew

**Figure 3:**
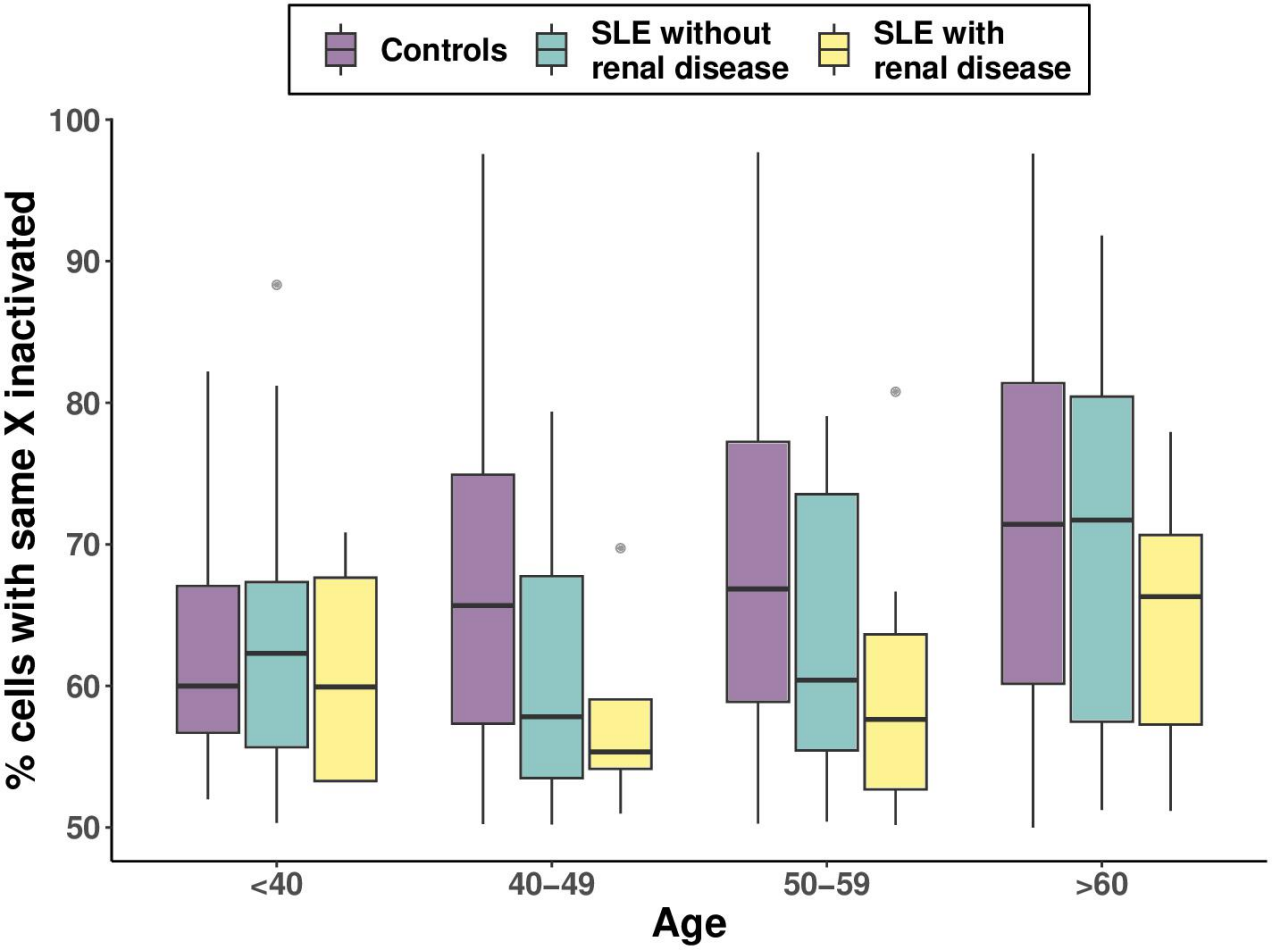
The effect of disease severity on XCI-skewing boxplots representing the association of renal disease as a marker of SLE severity on XCI skewing. All boxplots display the median and IQR, with XCI-skewing on the y-axis and age category on the x axis. SLE, systemic lupus erythematosus; XCI, X chromosome inactivation.

Given the association between SLE status and reduced XCI-skew, we hypothesised that a stronger effect would be observed in SLE cases with more severe disease, approximated by the presence of renal disease, which is associated with higher mortality and morbidity. To test this, we stratified the SLE cases based on a history of renal disease and compared each group (renal +ve and renal −ve) to the healthy controls. We observed a greater effect size on XCI-skew in those with renal disease (n=37; beta=−0.072; p=2.4×10^−4^) compared with those without renal disease (n=144; beta=−0.036; p=1.4×10^−3^; figure 3). Next, we compared which model was a better fit for the data. The first model included a binary variable which captured SLE status (controls/cases). The second model included a categorical variable which stratified the SLE cases by renal disease status (controls/renal −ve cases/renal +ve cases). We found the second model was nominally a better fit for the data (p=0.092). We also observed a nominally significant association between renal disease and reduced XCI-skew within the SLE samples only (beta=−0.031; p=0.091). Though the SLE patients with renal disease had a significantly higher mean age (56.7 years) compared with those without renal disease (47.3 years; Welch two sample t-test: p=6.5×10^−4^), only 1 individual (2.7%) with renal disease displayed a skewed XCI pattern (XCI≥80) compared with 11 individuals (7.6%) without renal disease (online supplemental figure S2).

### Medication use does not explain the case–control differences in XCI

We hypothesised that disease treatment might be driving the differences between cases and controls. Using the same approach as carried out for renal disease, we stratified the SLE patients based on use of hydroxychloroquine, methotrexate, azathioprine/mycophenolate and biologics and observed no difference in effect size between groups compared with controls (online supplemental figure S3; online supplemental table 1), suggesting medication use does not explain the difference in XCI between cases and controls. We also saw no significant effect of mediation use on XCI-skew within the SLE samples only (online supplemental table 1).

### Immune cell type composition does not explain the case–control differences in XCI

SLE manifests with significant changes to the abundance of cell populations in the peripheral blood and we postulated that cell composition could explain differences in XCI between cases and controls. As expected, we observed stark differences in full blood count data between cases and controls, with lower levels of lymphocytes and higher levels of monocytes and neutrophils in the SLE patients (online supplemental table 2). Given the strong correlation between SLE status and immune cell counts, it was not possible to add cell counts as covariates directly to the model. Instead, for each cell type in turn (n=7), we first regressed out their effects on XCI-skew (see the ‘Methods’ section) and found SLE status was still significantly associated with the residuals of XCI-skew following the removal of the effects of each cell type (online supplemental table 3). Of note, controlling for monocyte count, which we have previously reported to be positively associated with XCI-skew in a population cohort,^8^ augmented the effect of SLE status on XCI-skew (online supplemental figure S4). Therefore, the differences in cell proportions between cases and controls do not explain the XCI association.

### Genetic susceptibility to SLE is not associated with XCI-skew

Genome-wide association studies (GWASs) have identified many common genetic variants which increase the risk of developing SLE and the additive effect of the disease-associated variants can be captured using a PGS.^33^ We calculated the SLE-PGS across the cases and controls with available date (n=899) and demonstrated it is significantly associated with SLE status (online supplemental figure S5) p=0.001, OR=1.35 (95% CI 1.13 to 1.62)). However, we see no association between the SLE-PGS and XCI-skew (p=0.37, beta=0.0036), suggesting the inherited genetic susceptibility to SLE so far identified through GWAS does not influence XCI (online supplemental figure S6).

### Interferon signalling is associated with reduced XCI-skew in an age-dependent manner

**Figure 4:**
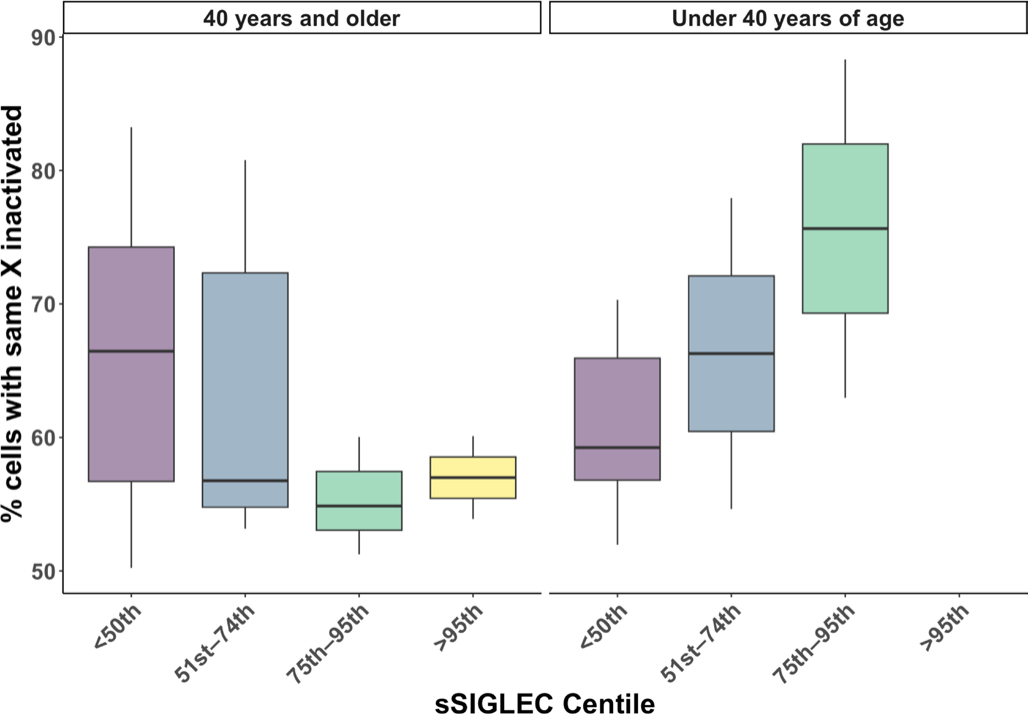
The age-dependent effects of IFN-signalling on XCI-skewing boxplots representing the association of non-linear sSIGLEC-1 percentile groups on XCI-skewing. All boxplots display the median and IQR, with XCI-skewing on the y-axis, and percentile groups on the x-axis. XCI, X chromosome inactivation.

We postulated that the effects of chronic interferon (IFN) signalling, a key hallmark of lupus pathology, could be the mechanism underpinning the disease effects on XCI-skew. We tested this hypothesis using measures of soluble SIGLEC-1 (sSIGLEC-1), a plasma biomarker for type I interferon-regulated gene expression.^31^ We observed a non-linear relationship between age and sSIGLEC-1 (online supplemental figure S7), and therefore, assessed the association between sSIGLEC-1 and XCI-skew using age as an interaction term (XCI-skew−sSIGLEC-1×age). We found this model was a better fit for the data (p=0.004) compared with the model with age as a covariate (XCI-skew−sSIGLEC-1+age). We observed an age-dependent association between sSIGLEC-1 and XCI-skew (n=41; p=0.009), with a significant interaction with age (p=0.004; figure 4).

## Discussion

The prevalence of XCI-skew in HSPC-derived immune cells increases with age and represents a common age-acquired phenotype in females.^10 32^ It has been hypothesised that XCI-skew of immune cells may play a causal role in the development and sex biases of autoimmune disease, with inadequate thymic deletion being the proposed mechanistic driver.^13^ Here, to the best of our knowledge, we present the largest study of XCI in SLE to date and demonstrate reduced XCI-skew in SLE cases compared with healthy controls, thus refuting the hypothesis that XCI-skew contributes to the sex bias of SLE.^13^

Instead, our results, which demonstrate that disease severity impacts XCI-skew, whereas differential WCC, medication or genetic susceptibility to SLE do not, suggest that the disease state itself is affecting XCI-skew. Further, we demonstrated an age-dependent association between XCI-skew and sSIGLEC-1, a plasma biomarker for type I interferon (IFN-I)-regulated gene expression, a key hallmark of lupus pathogenesis. We propose a mechanism in which the persistent IFN-I signature common in SLE impacts the HSPCs, which results in the XCI-skew differences compared with a control population. Both stem cell exhaustion and clonal expansion have been hypothesised as mechanisms underlying the XCI-skew signature commonly observed in ageing females.^10^ Though the role of interferons on the homeostasis of HSPCs is complex,^23 24 34^ the IFN-I signature in SLE has been shown not to cause stem cell exhaustion.^35 36^ Indeed, mice with active lupus have been shown to have HSCs with enhanced self-renewal capacity.^25^ Such an effect in SLE patients, driven by chronic IFN-I stimulation, could prevent stem cell exhaustion and maintain balanced XCI ratios in peripheral blood cells. These results reveal important insights into the ageing immune system in individuals with lupus and warrant follow-up studies across other autoimmune diseases and interferonopathies.

The age-dependent effects of sSIGLEC-1 on XCI-skew raise important considerations, which warrant further investigation. Specifically, older females are more likely to have had lupus for longer, and therefore, the effects of chronic IFN signalling could be more pronounced. Notably, it is at older ages that we expect to see a higher prevalence of XCI-skewing, and indeed we observed no difference in XCI-skew between cases and controls in the under 40s, therefore, this also could be an issue of power. It is important to note we have very limited numbers of younger patients with higher sSIGLEC scores.

Our work contrasts with earlier studies which also measured XCI using the HUMARA method and reported increased XCI-skew in autoimmunity, including rheumatoid arthritis,^16 17^ systemic sclerosis^18 19^ and autoimmune thyroid disease.^14^,^16^ Given these discrepant findings across autoimmune disease, it will be of great interest to establish whether XCI points to mechanistic differences in the development or effect of specific diseases, or indeed whether analytical methods underpin the different results. With this latter point in mind, it is of paramount importance that age is fully controlled for in case–control analyses due to the strong effect of ageing on XCI. Of note, some studies which report case–control differences in XCI did not observe the expected positive correlation between XCI-skew and age in the healthy control samples.^15 16 19^ In addition to controlling for age in every regression model, we ensured that our findings were not spuriously driven by differences in the age distribution of the cases and controls in two important ways. First, we validated the association within age strata (<40 years, 40–49 years, 50–59 years and ≥60 years). Second, we replicated our study using a small, independent cohort of SLE-discordant twin pairs who are perfectly matched for age.

As well as replicating our findings, the discordant twin study revealed an intriguing result: the discordant DZ twins had significant differences in XCI, whereas the MZ twins did not. This difference in DZ and MZ twins is in line with two previous studies. First, a small study in MZ twins discordant for SLE found no difference in XCI skew.^37^ Second, a twin study of XCI and serum levels of autoantibodies to thyroid peroxidase, a measure of subclinical thyroid disease, found differences in XCI between DZ twin pairs but not MZ twin pairs.^38^ The authors hypothesised that the findings could suggest XCI is not a causative factor in levels of autoantibodies to thyroid peroxidase, but instead that the two phenotypes could be driven by the same genetic confounders.^38^ Likewise, our findings suggest that XCI and SLE may be affected by the same underlying genetic factors. However, we observe no effect of the PGS for SLE on XCI. Of note, a limitation of PGS, and indeed GWAS more broadly, is that they typically only capture the effects of common single nucleotide variants. It is plausible that other sources of genetic variation, such as copy number variation or rare variants, could underpin unexplained genetic effects.

XCI is initiated by the long non-coding RNA *XIST* which acts in cis to recruit protein complexes and epigenetic changes to silence the inactivated X (Xi). Functional studies have demonstrated a dynamic role of XIST during the development and maturation of T and B cells, where XIST is no longer localised to the Xi in naïve cells.^39 40^ Further, this dynamic role of XIST has been demonstrated to be disrupted in both a mouse model of SLE and human SLE patients, suggestive of impaired transcriptional regulation of the X chromosome in lymphocytes as a feature of SLE.^39 41^ Crucially, DNA methylation, which is required for the epigenetic memory of the Xi to be maintained throughout cell divisions, is not thought to be disrupted during loss of XIST RNA localisation.^40 42^ The HUMARA assay is dependent on the methylation of the *AR* locus on the Xi, and therefore, would not be impacted by the dysregulation of XIST in SLE.

Our study does have limitations. The discordant twin study was of limited sample size. However, such discordant twin models are powerful. For some phenotypes, notably whole blood count data and the sSIGLEC-1 data, we had a high percentage of samples with missing data. This prevented us from carrying out further analyses, such as controlling for the effects of cell composition when testing differences between those with and without renal disease. Likewise, we lacked sufficient data to assess molecular and cellular markers of disease activity, which may have informed our interpretation. Of note, work by our group has previously reported no correlation between C reactive protein and XCI-skew.^8^

In summary, we have demonstrated that XCI-skew is reduced in SLE cases compared with healthy controls drawn from a population cohort. We postulate that chronic IFN-I signalling impacts HSPCs, and this is reflected in the XCI patterns of HSPC-derived immune cells. Further work is needed to confirm this mechanism, which could reveal important insights into the ageing immune system in individuals with autoimmunity.

## supplementary material

10.1136/ard-2024-225585 online supplemental file 1

## Data Availability

Data are available on reasonable request.
