# Supplementary material for: Haematopoietic stem cell-derived immune cells have reduced X chromosome inactivation skewing in systemic lupus erythematosus
Source: Ann Rheum Dis. 2024 Jun 27;83(10):e225585. doi: 10.1136/ard-2024-225585 (PMC11503196; doi:10.1136/ard-2024-225585)

Supplementary

Supplementary Table 1: Results of association of medication use on XCI-skewing

The beta columns refer to the effect size of those with/without the drug compared to controls. “P value models” refers to the fit of the model with medication use as a categorical variable compared to the model with SLE as a binary variable. “P value SLE” refers to the P value of the effect of the medication within the SLE cohort only.

**Measure**	**Beta +ve**	**Beta -ve**	**P value models**	**P value SLE**
Hydroxychloroquine	-0.040	-0.045	0.81	0.97
Methotrexate	-0.014	-0.049	0.16	0.10
Azathioprine or mycophenolate	-0.028	-0.048	0.33	0.36
Biologics	-0.035	-0.044	0.80	0.82

Supplementary Table 2: Immune cell differences between SLE cases and healthy controls

P-values refer to the effect of SLE status on the cell count/measure as taken from a linear regression model controlling for age as a covariate.

**Measure**	**Controls (mean)**	**SLE Cases (mean)**	**P value**
White Blood Cells	5.84	6.32	0.010
Neutrophils	3.51	4.24	0.0027
Monocytes	0.41	0.54	5.43e-10
Lymphocytes	1.74	1.39	6.31e-05
Lymphocyte Proportion	0.30	0.23	3.03e-09
Monocyte-to-Lymphocyte Ratio	0.25	0.52	2.73e-25
Neutrophil-to-Lymphocyte Ratio	2.17	4.15	9.78e-12

Supplementary Table 3: Results of association of SLE status on XCI-skewing following removal of effects of immune cell counts.

**Measure**	**Beta**	**P value**
White Blood Cells	-0.049	0.013
Neutrophils	-0.048	0.013
Monocytes	-0.051	0.0092
Lymphocytes	-0.043	0.028
Lymphocyte Proportion	-0.047	0.015
Monocyte-to-Lymphocyte Ratio	-0.044	0.025
Neutrophil-to-Lymphocyte Ratio	-0.044	0.025

Supplementary Figure 1: XCI-skewing is reduced in SLE cases across age groups. Box plots representing the association of SLE on XCI skewing. All boxplots display the median and IQR, and XCI-skewing on the y-axis, and age category on the x axis.

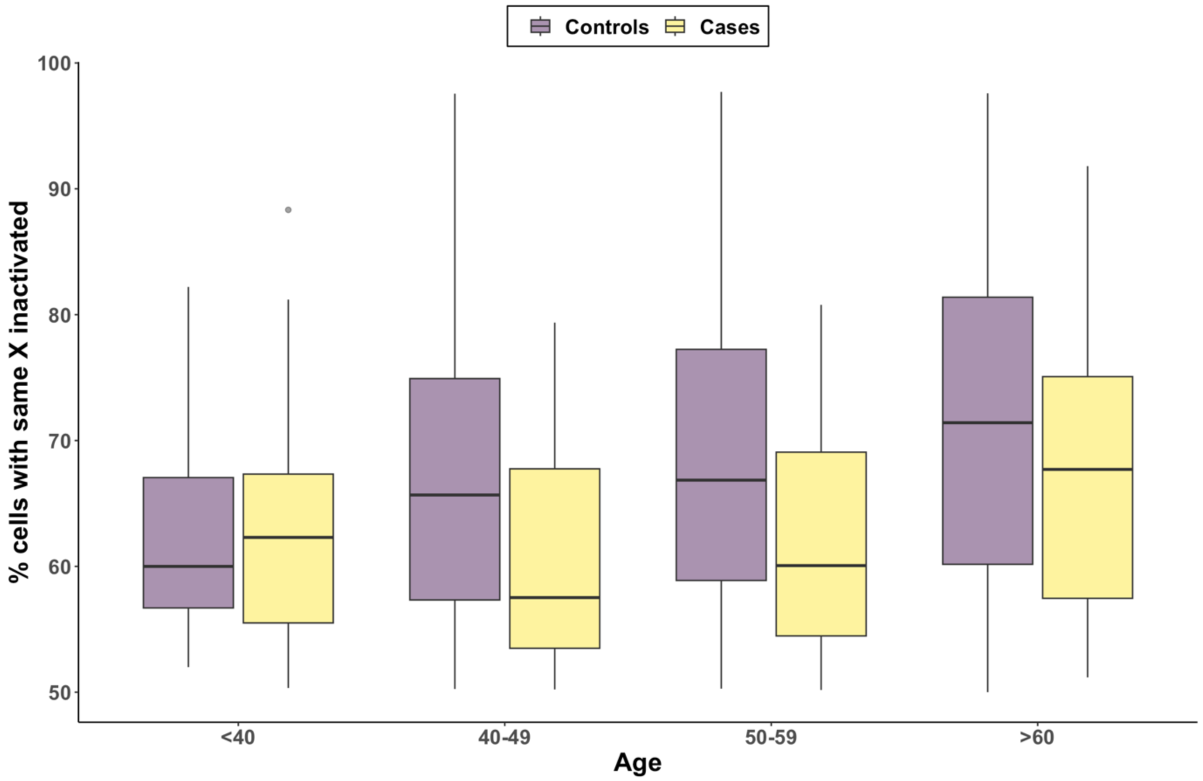

Supplementary Figure 2: XCI-skewing in SLE cases with and without renal disease compared to controls.

The correlation between XCI (y-axis) and age (x-axis) is shown in panels on the left and the proportions of individuals (y-axis) with random (50-79%), skewed (80-89%), and extremely skewed (>90%) XCI across increasing age groups (x-axis) are shown in panels on the right. Controls (n=796) are in the upper panels, SLE cases without a history of renal disease (n=144) are in the middle panel, and SLE cases with a history of renal disease (n=37) are in the lower panels.

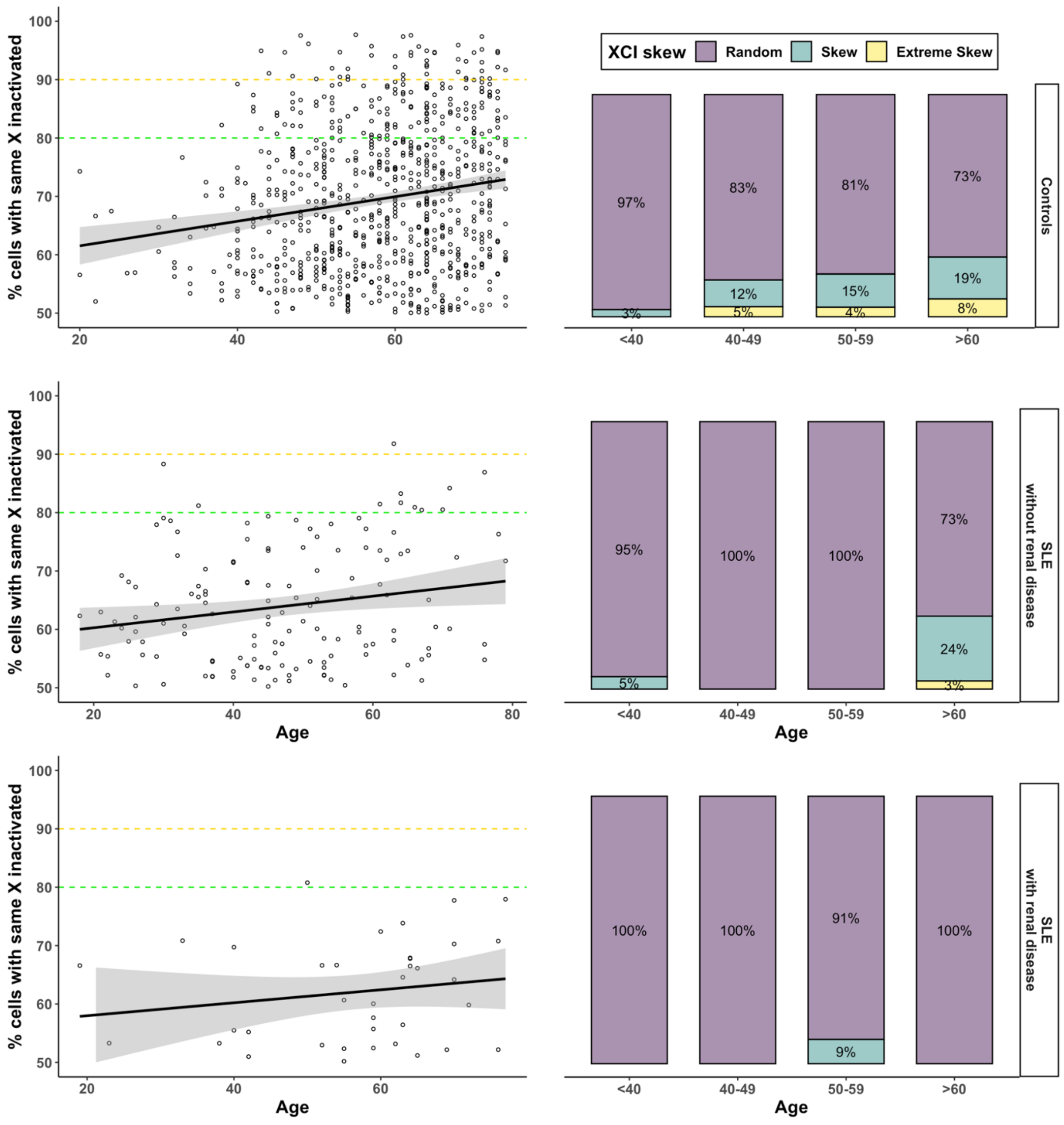

Supplementary Figure 3: The effect of medication on XCI skewing.

Box plots representing the effect of medication use on XCI skewing. All boxplots display the median and IQR, and XCI-skewing on the y-axis, and age category on the x axis.

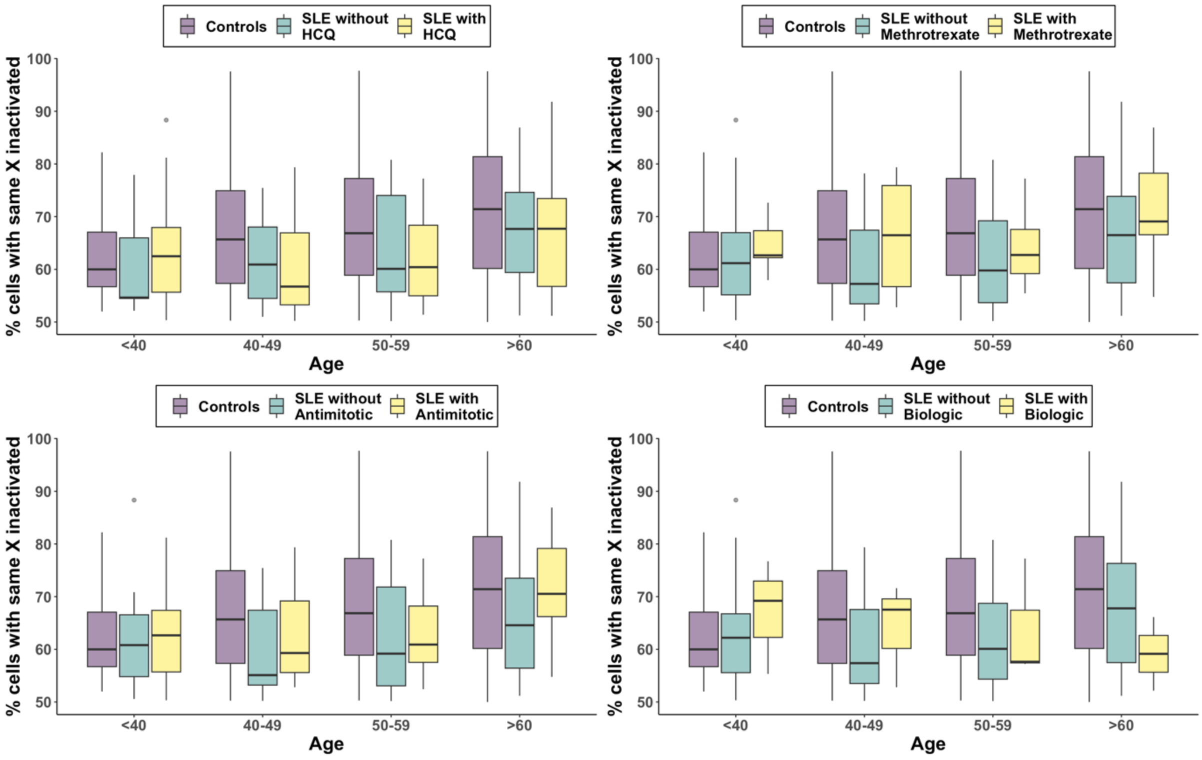

Supplementary Figure 4: XCI-skewing is reduced in SLE cases across age groups after regressing out effects of Monocyte counts.

Box plots representing the association of SLE on XCI skewing. All boxplots display the median and IQR, and XCI-skewing on the y-axis, and age category on the x axis.

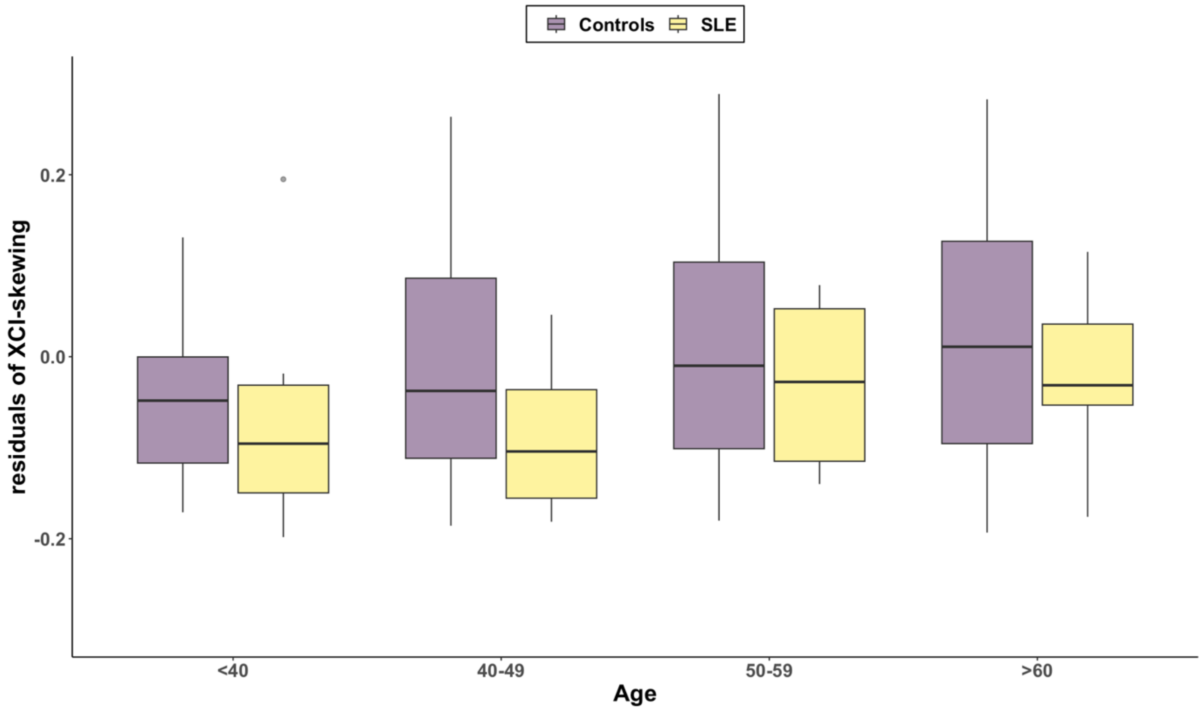

Supplementary Figure 5: Polygenic Risk Score for SLE is associated with SLE status

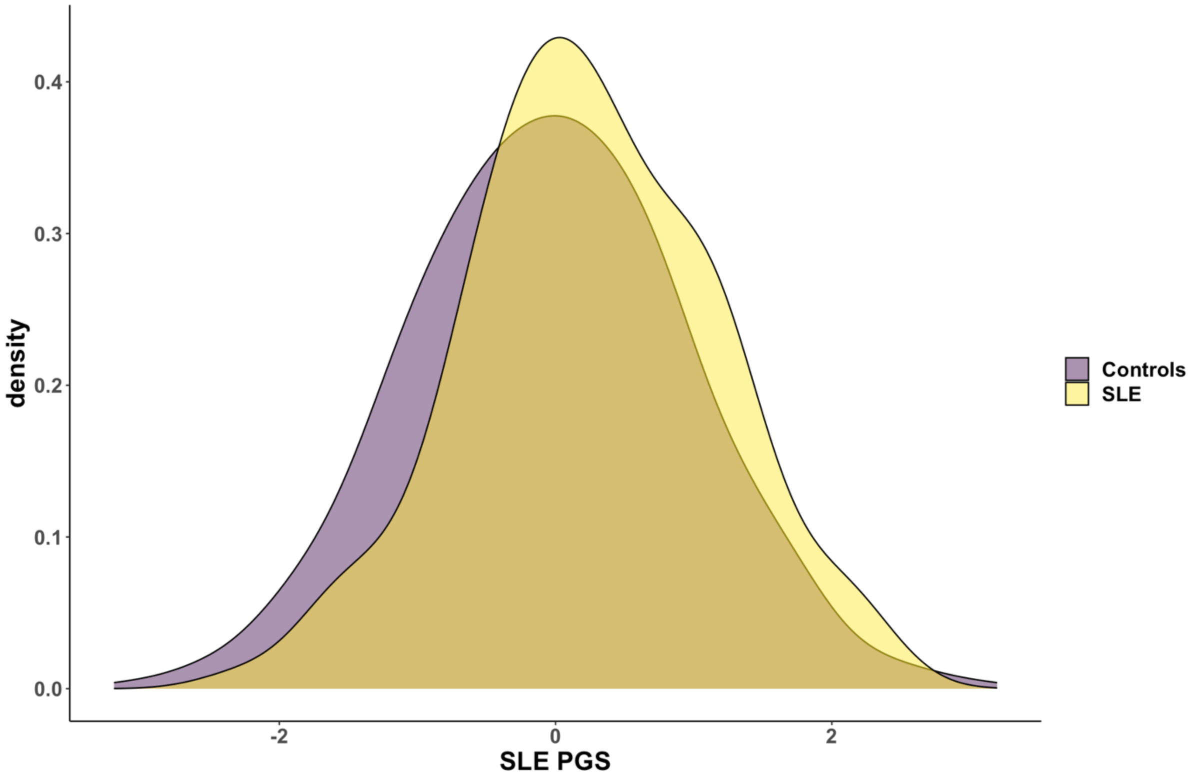

Supplementary Figure 6: No association of SLE Polygenic Risk Score and XCI-skewing

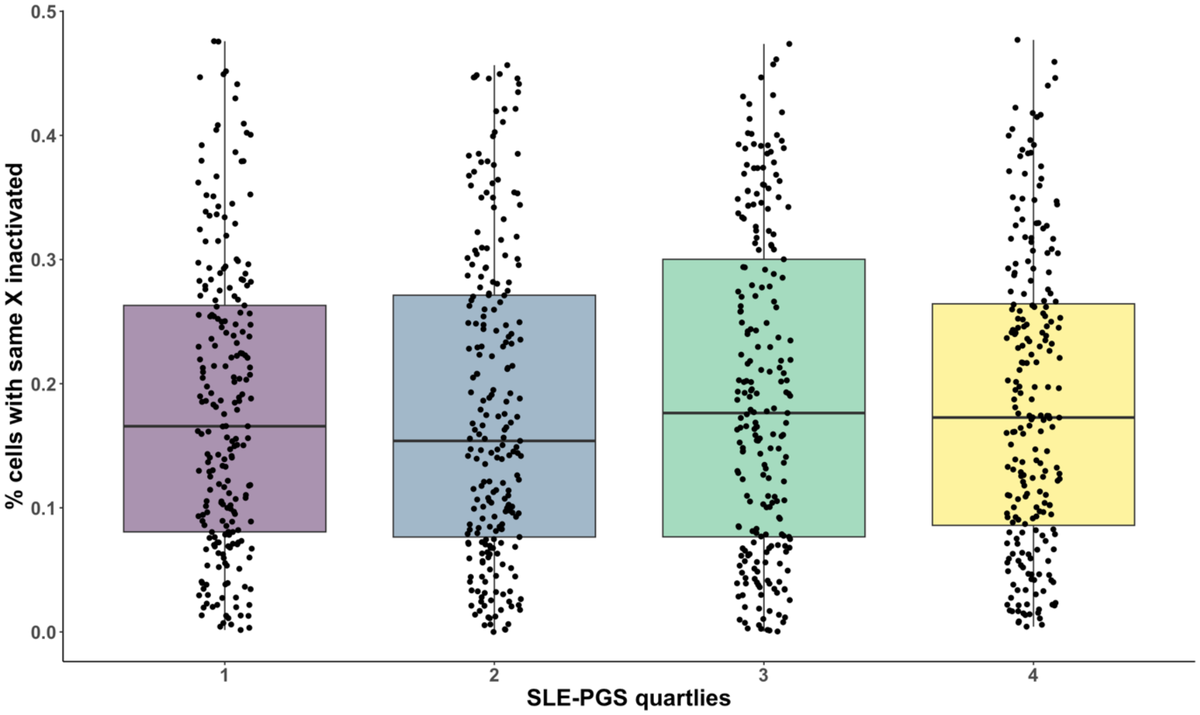

Supplementary Figure 7: A non-linear relationship between age and sSIGLEC-1

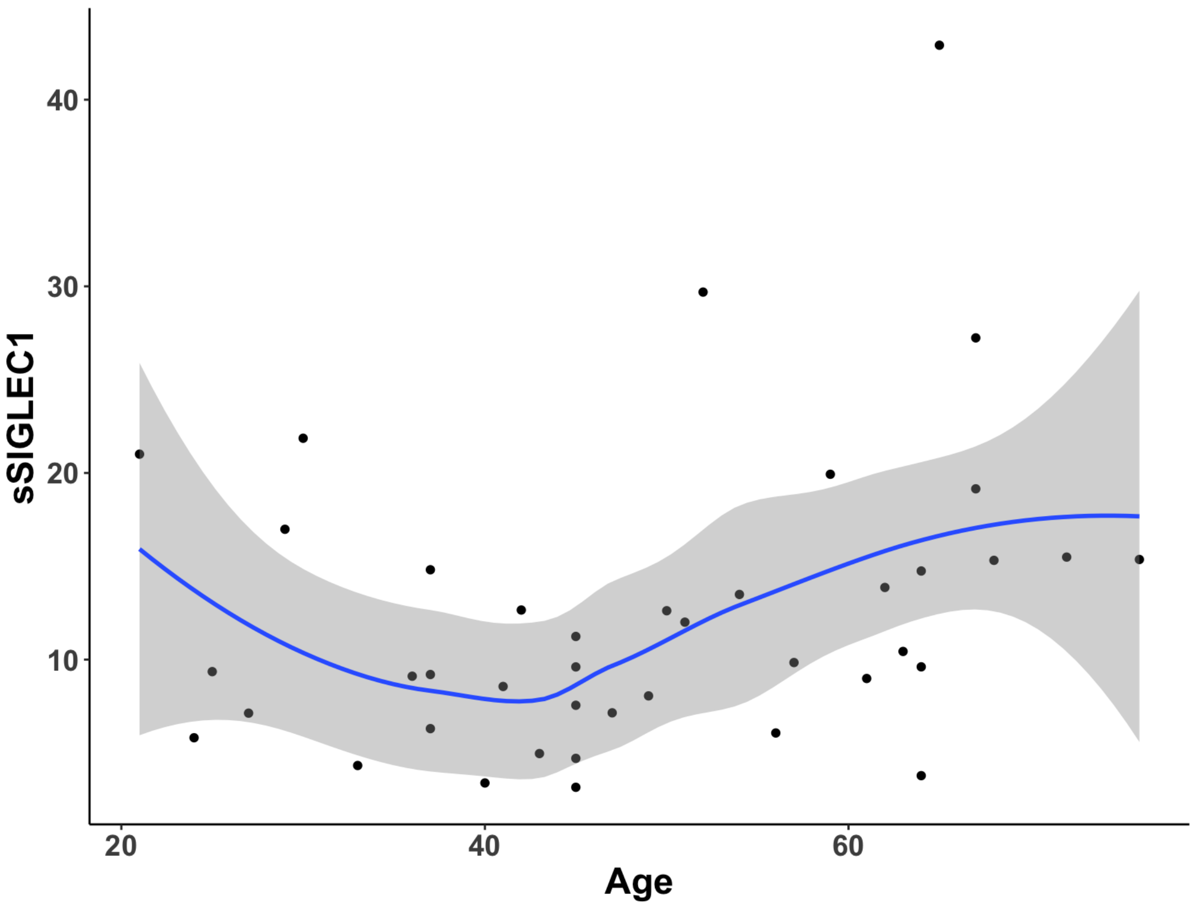

Supplement: online supplemental file 1 [file ard-83-10-s001.pdf]
